# Course of symptoms for loss of sense of smell and taste over time in one thousand forty‐one healthcare workers during the Covid‐19 pandemic: Our experience

**DOI:** 10.1111/coa.13683

**Published:** 2020-12-21

**Authors:** Matt Lechner, Jacklyn Liu, Nicholas Counsell, Ngan Hong Ta, John Rocke, Rajesh Anmolsingh, Nicholas Eynon‐Lewis, Santdeep Paun, Claire Hopkins, Sadie Khwaja, B. Nirmal Kumar, Samuel Jayaraj, Valerie J. Lund, Carl Philpott

**Affiliations:** ^1^ Barts Health NHS Trust London UK; ^2^ UCL Cancer Institute University College London London UK; ^3^ Cancer Research UK & UCL Cancer Trials Centre University College London London UK; ^4^ The Norfolk Smell & Taste Clinic Norfolk & Waveney ENT Service Norfolk UK; ^5^ Norwich Medical School University of East Anglia Norwich UK; ^6^ Wrightington, Wigan and Leigh Teaching Hospitals NHS Foundation Trust London UK; ^7^ Manchester University NHS Foundation Trust London UK; ^8^ Guy’s and St. Thomas’ NHS Foundation Trust London UK; ^9^ Royal National Throat, Nose and Ear Hospital UCLH Foundation Trust London UK

**Keypoints**

- Questionnaires were distributed at six NHS trusts; 1041 individuals completed the questionnaires between 27th March and 9th June 2020.
- Nearly two‐thirds of participants reported recent sudden loss of sense of smell and/or taste.
- Loss of sense of smell and/or taste was significantly associated with a positive Covid‐19 test.
- For those in who loss of smell and/or taste occurred at least 4 weeks prior to the survey, only half had fully recovered, indicating the need for further research into the long‐term management of the sequelae of Covid‐19 infection.
- Increased awareness and recognition of these symptoms are crucial, in particular amongst healthcare personnel who need to be urgently tested and to self‐isolate accordingly in order to reduce spread.

Dear Editor,

On 21 April 2020, the Centers for Disease Control and Prevention (CDC) and on 5 May 2020, the World Health Organisation added “new loss of taste or smell” to their list of symptoms related to Covid‐19, respectively. Public Health England (PHE) only included loss of smell and taste as official symptoms on 20th May. However, whether individual hospitals were including smell and taste disturbances in their initial work‐up for Covid‐19 diagnosis is unknown.

Several reports have now demonstrated the prevalence of these symptoms in Covid‐19 infected individuals with important implications on diagnosis and management.^1^ Due to the high incidence of Covid‐19 infection which has been reported in the healthcare setting, we sought to understand the symptomatology of a cohort of healthcare personnel in order to assess current management guidelines, including how many healthcare workers were permitted to continue working despite being infected.

## 1 MATERIALS AND METHODS

### 1.1 Survey design and recruitment

Anonymous questionnaires were distributed to staff at hospitals across six NHS trusts: Barts Health, Guy's and St. Thomas’, James Paget University Hospital, Norfolk & Norwich University Hospital, Manchester University and Wrightington, Wigan and Leigh. The study was advertised by trust‐wide emails to all staff, which included access to an electronic link to the questionnaire. Participants completed the questionnaire online (supplementary material).

Slight alterations to the survey format were utilised, due to logistical and practical reasons across the various trusts. Results from all trusts were pooled at the end of the recruitment period. Questionnaires were distributed and completed between 27 March 2020 and 9 June 2020. A single questionnaire was distributed to three of the six trusts, whilst two questionnaires (baseline and follow‐up) were distributed to the remaining three trusts. Question items were largely identical between the two methodologies; where different, questions were appropriately matched for analysis. This pertains to questions regarding whether participants had experienced the loss of sense of smell/taste within the past 4 weeks and whether or not they had recovered, which was either explicitly asked in the single questionnaire format or implied in the second methodology, on the basis of the baseline and follow‐up questionnaires being completed 4 weeks apart. In addition, where relevant, only those who completed the follow‐up survey were asked about qualitative smell dysfunction (ie distortion of smell, phantom smells and sensations of burning/cooling/tingling in nose and/or mouth). The remainder of the questionnaires included items on age, sex, Covid‐19 status and relevant symptoms and specific questions around chemosensory disturbances, such as a smell and taste rating, which were identical between the two methodologies.

Descriptive statistical analysis was conducted on patient characteristics and logistic regression where odds ratios (with 95% confidence intervals and *P*‐values) are presented. Smell and taste scores were compared between groups using the Mann‐Whitney *U* test. Sensitivity and specificity of the loss of sense of smell/taste in relation to Covid‐19 infection are also presented, in addition to positive and negative predictive values.

## 2 RESULTS

### 2.1 Participant characteristics

In total, 1041 healthcare workers from the six NHS trusts responded to our surveys from 27 March 2020 to 9 June 2020. Sample characteristics are described in Table 1; 70.9% female (737 of 1039), 74.6% white (456 of 611), 75.1% work in direct contact with patients (489 of 651), 50.3% (524 of 1041) were aged ≤ 40 years and 5.6% (58 of 1041) aged over 60 years. Overall, 306 (29.4%) participants had been tested for Covid‐19—208 (20.0% of total, 68.0% of tested) had a confirmed positive test and 98 (9.4% of total, 32.0% of tested) were negative; a further 315 (30.3%) had not been tested but suspected that they had been infected, and 420 (40.3%) did not suspect that they had been infected.

**Table 1:** Survey characteristics (N = 1041)

Characteristic/Question	Category	n (%)	
Age	30 or under	251 (24.1)	
	31‐40	273 (26.2)	
	41‐50	244 (23.4)	
	51‐60	215 (20.7)	
	Over 60	58 (5.6)	
Gender (n = 1039)	Female	737 (70.9)	
	Male	301 (29.0)	
	Non‐binary	1 (0.1)	
Ethnicity (n = 611)	White	456 (74.6)	
	Other	155 (25.4)	
NHS trust	Barts Health	273 (26.2)	
	Guy's and St Thomas'	86 (8.3)	
	James Paget University Hospitals	63 (6.1)	
	Norfolk & Norwich University Hospital	91 (8.7)	
	Manchester University	428 (41.1)	
	Wrightington, Wigan and Leigh	100 (9.6)	
Does your work involve direct patient contact? (n = 651)	No	162 (24.9)	
	Yes	489 (75.1)	
Covid‐19 Coronavirus status?	Negative tested	98 (9.4)	
	Negative opinion	420 (40.3)	
	Positive opinion	315 (30.3)	
	Positive tested	208 (20.0)	
TOTAL			1041 (100.0)

### 2.2 Overall symptomatology

In total, 62.3% (649 of 1041) of all participants reported losing their sense of smell/taste in the last 2 months, 16.6% of whom reported it as the only symptom (12 missing). Of the participants, who reported Covid‐19–related symptoms (with or without a positive test/suspicion of infection and excluding loss of smell/taste), the most common experienced were fatigue (65.4%), cough (42.5%) and fever (40.2%). 720 participants reported the severity of their symptoms where 54.6% were mild, 43.8% moderate and 1.7% severe. Overall, 385 of 793 (48.5%) had continued to work as normal during this time. Of the 272, who responded, 247 participants experienced at least one of the following symptoms: chemesthesis (ie sensations of burning, cooling or tingling) in nose or mouth (34.6%, 94 of 272), parosmia (42.3%, 115 of 272) and phantosmia (33.8%, 92/272).

### 2.3 Self‐reported experience with smell and taste dysfunction

There was strong evidence of an association between losing the sense of smell/taste and Covid‐19 (Table 2), participants who lost their sense of smell/taste were more likely to have a positive Covid‐19 test (Odds Ratio OR = 8.55, 95% CI: 4.69‐15.58, *P* < .001) or suspected Covid‐19 infection (OR = 14.55, 95% CI: 10.49‐20.18, *P* < .001), that is an 8½‐fold increase in risk within the tested subgroup or a 14½‐fold increase in risk when including both tested and non‐tested participants. The sensitivity and specificity of smell/taste loss being symptoms of Covid‐19 positive cases were 89.9% and 49.0%, respectively, whilst the positive predictive and negative predictive values were 78.9% and 69.6%, respectively (Table 3). Similar results were observed when considering the scores, participants gave with regard to their sense of smell/taste at its worst (0 none‐10 normal), with markedly lower scores in the Covid‐19 positive groups in terms of sense of smell (tested subgroup *P* < .001; all participants *P* < .001) and sense of taste (tested subgroup *P* = .05; all participants *P* < .001).

**Table 2:** Survey responses by Covid‐19 coronavirus status (N = 1041)

Question	N = 1041			Category		Negative tested	Negative opinion	Positive opinion	Positive tested	Total
	Not answered	Normal Smell/Taste	Not asked^a^							
Have you suddenly lost your sense of smell/taste in the last 2 mo? (n = 1041)	0 (0%)	NA	NA	No		48 (49.0%)	286 (68.1%)	37 (11.7%)	21 (10.1%)	392 (37.7)
				Yes		50 (51.0%)	134 (31.9%)	278 (88.3%)	187 (89.9%)	649 (62.3)
Have you noticed any of the following? (n = 272)	533 (51.2%)	NA	236 (22.7%)	Changes in the sensations of burning, cooling or tingling in your nose or mouth?		13 (13.8%)	19 (20.2%)	32 (34.0%)	30 (31.9%)	94 (34.6%)
				Distortion of smell (things smell differently to what you expect?)		15 (13.0%)	18 (15.7%)	53 (46.1%)	29 (25.2%)	115 (42.3%)
				Feeling like you are smelling something when there is no smell present?		10 (10.9%)	16 (17.4%)	40 (43.5%)	26 (28.3%)	92 (33.8%)
				None of the above		0 (0.0%)	5 (25.0%)	16 (64.0%)	4 (16.0%)	25 (9.2%)
How would you rate your sense of smell at its worst? (n = 767)	274 (26.3%)	NA	NA	mean (standard deviation), 0 none‐10 normal		3.4 (3.79)	4.5 (4.11)	1.6 (2.82)	1.2 (2.65)	2.5 (3.56)
				median (range) 0 none‐10 normal		1.0 (0‐10)	3.0 (0‐10)	0.0 (0‐10)	0.0 (0‐10)	0.0 (0‐10)
How would you rate your sense of taste at its worst? (n = 767)	274 (26.3%)	NA	NA	mean (standard deviation), 0 none‐10 normal		3.6 (3.44)	5.0 (3.95)	2.4 (2.91)	2.6 (3.05)	3.3 (3.50)
				median (range) 0 none‐10 normal		2.0 (0‐10)	4.0 (0‐10)	1.0 (0‐10)	2.0 (0‐10)	2.0 (0‐10)
If not ongoing, how long did loss of taste/smell last for? (n = 419)^b^	114 (11.0%)	392 (37.7%)	NA	mean (standard deviation), days		12.8 (12.97)	14.8 (12.86)	16.9 (13.12)	15.4 (11.26)	15.7 (12.60)
				median (range), days		10.0 (1‐60)	10.0 (1‐60)	14.0 (1‐90)	12.0 (1‐60)	14.0 (1‐90)
Did your sense of smell/taste disappear...? (n = 637)	12 (1.2%)	392 (37.7%)	NA	Only symptom		8 (7.6%)	57 (53.8%)	23 (21.7%)	18 (17.0%)	106 (16.6%)
				Before others		9 (9.2%)	13 (13.3%)	44 (44.9%)	32 (32.7%)	98 (15.4%)
				After others		32 (7.4%)	61 (14.1%)	208 (48.0%)	132 (30.5%)	433 (68.0%)
If you have suffered symptoms were they...? (n = 720)	321 (30.8%)	NA	NA	Mild but continued to work		1 (2.2%)	25 (54.3%)	16 (34.8%)	4 (8.7%)	46 (6.4)
				Mild and self‐isolate		50 (14.4%)	89 (25.6%)	123 (35.4%)	85 (24.5%)	347 (48.2)
				Moderate		27 (8.6%)	39 (12.4%)	150 (47.6%)	99 (31.4%)	315 (43.8)
				Severe		1 (8.3%)	0 (0.0%)	3 (25.0%)	8 (66.7%)	12 (1.7)
Have you…? (n = 793)	248 (23.8%)	NA	NA	Needed treatment in hospital		0 (0.0%)	0 (0.0%)	0 (0.0%)	3 (100.0%)	3 (0.4%)
				Self‐isolated		47 (12.1%)	53 (13.7%)	159 (41.0%)	129 (33.2%)	388 (48.9%)
				Partially self‐isolated		3 (17.6%)	5 (29.4%)	5 (29.4%)	4 (23.5%)	17 (2.1%)
				Continued to work as normal		36 (9.4%)	234 (60.8%)	88 (22.9%)	27 (7.0%)	385 (48.5%)
What other symptoms have you experienced? (n = 767)	274 (26.3%)	NA	NA	Nasal blockage		21 (8.3%)	61 (24.2%)	102 (40.5%)	68 (27.0%)	252 (32.9)
				Runny nose		22 (11.0%)	53 (26.5%)	68 (34.0%)	57 (28.5%)	200 (26.1)
				Nasal irritation/burning		10 (8.3%)	18 (15.0%)	48 (40.0%)	44 (36.7%)	120 (15.6)
				Distortion of smell		7 (13.7%)	8 (15.7%)	21 (41.2%)	15 (29.4%)	51 (6.6)
				Bad smell		5 (8.6%)	14 (24.1%)	18 (31.0%)	21 (36.2%)	58 (7.6)
				Fever		27 (8.8%)	35 (11.4%)	147 (48.1%)	99 (32.1%)	308 (40.2)
				Cough		22 (6.7%)	43 (13.2%)	157 (48.2%)	104 (31.9%)	326 (42.5)
				Diarrhoea		17 (11.6%)	15 (10.2%)	66 (44.9%)	49 (33.3%)	147 (19.2)
				Shortness of breath		16 (7.0%)	29 (12.8%)	101 (44.5%)	81 (35.7%)	227 (29.6)
				Fatigue		37 (7.4%)	89 (17.7%)	222 (44.2%)	154 (30.7%)	502 (65.4)
Has it been ≥ 4 wk since you lost sense of smell/taste? (n = 519)	3 (0.3%)	392 (37.7%)	127 (12.2%)	No		11 (18.0%)	5 (8.2%)	12 (19.7%)	33 (54.1%)	61 (11.8%)
				Yes		35 (7.6%)	93 (20.3%)	214 (46.7%)	116 (25.3%)	458 (88.2%)
Has your sense of smell and/or taste now recovered? (n = 496)	26 (2.5%)	392 (37.7%)	127 (12.2%)	≥4 wk (n = 455)	No	6 (13.3%)	6 (13.3%)	16 (35.6%)	17 (37.8%)	45 (9.9%)
					Yes, partially	9 (5.1%)	26 (14.8%)	95 (54.0%)	46 (26.1%)	176 (38.7%)
					Yes, completely	19 (8.1%)	61 (26.1%)	102 (43.6%)	52 (22.2%)	234 (51.4%)
				<4 wk (n = 41)	No	0 (0.0%)	0 (0.0%)	2 (25.0%)	6 (75.0%)	8 (19.5%)
					Yes, partially	5 (20.8%)	2 (8.3%)	4 (16.7%)	13 (54.2%)	24 (58.5%)
					Yes, completely	3 (33.3%)	1 (11.1%)	2 (22.2%)	3 (33.3%)	9 (22.0%)

Abbreviation: NA, not applicable.

^a^ Did not complete follow‐up questionnaire.

^b^ Excludes 116 participants who reported ongoing symptoms.

**Table 3:** The loss of sense of smell and/or taste in predicting a positive Covid‐19 test

	Tested positive	Tested negative
*Reported loss of smell/taste*	187	50
*Did not report loss of smell/taste*	21	48
**Sensitivity** **Specificity** **Positive predictive value** **Negative predictive value**	**89.9%** **49.0%** **78.9%** **69.6%**	

Bold highlights the importance of smell loss as a symptom in Covid‐19.

In 519 participants who had recently lost their sense of smell and responded to the question: “has it been 4 weeks since you lost your sense of smell/taste?” 88.2% (458 of 519) reported that it had been at least 4 weeks since they had lost their sense of smell/taste—9.9% (45 of 455) had not yet recovered, 38.7% (176 of 455) had recovered partially and 51.4% (234 of 455) had recovered completely (3 missing). Of those for whom loss of smell/taste occurred within the past 4 weeks (n = 61), 19.5% (8 of 41) had not yet recovered, 58.5% (24 of 41) had recovered partially and 22.0% (9 of 41) had recovered completely (20 missing). Of the total cohort, the median loss was 14 days (range: 1‐90), which excludes 116 participants with ongoing symptoms.

## 3 DISCUSSION

This study demonstrates the very high prevalence of the loss of sense of smell and/or taste in healthcare personnel, which is significantly related to Covid‐19 positivity. In addition, these symptoms may occur with or without the presence of other Covid‐19–related symptoms; in 16.6% of the cohort, the loss of sense of smell/taste was the only symptom. This indicates that a number of individuals deemed to be otherwise asymptomatic may in fact be highly contagious and should have been self‐isolating.

Importantly, our results demonstrate that nearly half of participants continued to work following the loss of sense of smell and/or taste. Some participants noted that when seeking advice regarding self‐isolation from health authorities, they were told to continue working as loss of sense of smell and/or taste had yet to be recognised as official symptoms of Covid‐19 (qualitative data are not shown). Thus, it is likely that a large proportion of healthcare workers, who did continue to work, were in fact highly contagious at the time.

Interestingly, whilst objective smell testing was beyond the scope of this study, we do note that symptom self‐reporting (ie smell and taste rating) correlated with Covid‐19 positivity. This has been similarly demonstrated by a group at King's College London, where symptom tracking using a mobile app demonstrated that self‐reporting of the loss of sense of smell, in addition to fatigue, cough and loss of appetite, may more accurately predict Covid‐19 infection.^2^ Previously, the correlation between subjective and objective assessment of smell function has been shown to be poor.^3^ However, more detailed questioning can improve the sensitivity of self‐reported smell function as demonstrated by the US National Health and Nutrition Examination Survey (NHANES).^4^ Thus, whilst we cannot conclude whether self‐reporting through our questionnaire can predict objective testing outcome, subjective assessment as an initial screen may be beneficial in the detection of Covid‐19, especially given the typically sudden onset of the symptom, which is not as characteristic in olfactory dysfunction caused by other viruses. Efforts toward a validated, comprehensive questionnaire, which does correlate better with objective testing, would further this endeavour.

Although both loss of sense of smell and taste improved over time in the majority, there is a need to address the long‐term effects of these symptoms, as nearly half of those who lost their sense of smell/taste at least 4 weeks prior to the survey had yet to recover at the time of the survey. The reported persistence of smell/taste disturbances requires improved management guidelines, and points to the need for more treatments to be evaluated through clinical trials. At present, guidelines published by ENT UK/British Rhinological Society recommend those with recent loss of smell and Covid‐19 infection are managed by their GP with treatments suggested, which may include oral steroids after the initial 2 weeks.^5^ Further guidance can be found on supportive resources such as those available through Fifth Sense (www.fifthsense.org.uk).

A limitation to self‐reported questionnaires in general is the potential for response/selection and recall biases. Furthermore, since a repeat questionnaire was used at three sites, as opposed to the singular questionnaire was used at the remaining sites, it was not possible to perfectly match the questions and subsequent results. In addition, the design of the study allowed participants to opt out of certain questions. As such, there is necessarily missing data, which may impact the results.

In addition, a further limitation of this study is that only 20% of respondents had proven Covid‐19. An additional 30% of respondents reported a suspicion of Covid‐19 infection. Whilst Covid‐19 infection in the latter group could not be confirmed, it is highly likely these individuals suffered from infection as the chance of sudden loss of smell and/or taste not due to Covid‐19 infection is extremely rare. Indeed, a recent study demonstrated a positive rate of 87.5% for those who are tested within the first 12 days of isolated sudden loss of smell, further indicating that those who experience this isolated symptom are likely to have Covid‐19 infection.^5^

Moreover, it is important to note that, at the time of the study, the loss of sense of smell and/or taste had only recently emerged as potential cardinal symptoms of Covid‐19 infection. Therefore, Covid‐19 testing was not readily available, nor at times recommended, for those experiencing these symptoms, as they had not at that time been recognised by Public Health England. As such, discounting self‐reported Covid‐19 infection would certainly provide a limited evaluation of the population of healthcare workers being evaluated in this particular context.

Ultimately, we believe that our results provide important further information and context to the current status of Covid‐19 disease in a healthcare setting. Whilst we cannot estimate the true prevalence of smell and taste disturbance due to Covid‐19, the high proportion reported here is in line with other large datasets reliant on self‐reporting such as the Global Consortium for Chemosensory Research questionnaire.^2^, ^6^, ^7^, ^8^, ^9^ In contrast, a study in South Korea, which assessed only individuals who had a positive Covid‐19 test, found that roughly 30% of patients with early stage or mild disease experienced anosmia/ageusia.^10^ Nevertheless, whilst the true proportion of those who experience the loss of sense of smell/taste may be lower than observed in our study, our results indicate the importance of these symptoms and demonstrate their utility as part of targeted mass screening.

It has been evident that healthcare workers frequently report loss of sense of smell, particularly in the absence of other symptoms and looking at other studies, it can now be regarded likely that most healthcare workers, who have suffered from a loss of sense of smell at the time of the first wave of the pandemic, are likely to have had Covid‐19 infection as the chance of sudden loss of olfactory function is so rare otherwise, especially with limited social contact in a national lockdown. Therefore, in order to prevent hospital transmission, it is imperative that the 20% rate of testing seen in the first wave must be improved upon, particularly as Europe faces a second wave of infection, which may be greater than the first. Increased awareness of Covid‐19 symptomatology, in particular the loss of sense of smell, and implementation of due measures will certainly help to mitigate the increasing severity of the ongoing crisis.

## CONFLICT OF INTEREST

None to declare.

## Supporting information

Supplementary Material

## Data Availability

The data that support the findings of this study are available from the corresponding author upon reasonable request.
