# Supplementary material for: Course of symptoms for loss of sense of smell and taste over time in one thousand forty‐one healthcare workers during the Covid‐19 pandemic: Our experience
Source: Clin Otolaryngol. 2020 Dec 21;46(2):451–7. doi: 10.1111/coa.13683 (PMC8240100; doi:10.1111/coa.13683)
Supplement: Supplementary file 1 — Supplementary Material [file COA-46-451-s001.docx]

**Supplemental Material 1.** Initial Questionnaire and Follow-Up Questionnaire

*Initial Questionnaire*

1. Date of survey completion
2. Have you completed this survey previously? If yes, please add date of previous entry in ‘any other information’ at the bottom.

☐ Yes

☐ No

1. What is your age group?

☐ Under 30

☐ 31 – 40

☐ 41 – 50

☐ 51 – 60

☐ Over 60

1. What is your gender?

☐ Male

☐ Female

☐ Non-binary

☐ Prefer not to say

1. Have you been tested for Covid-19 Coronavirus?

☐ Yes and positive

☐ Yes and negative

☐ No but suspected positive

☐ No and no current suspicion

1. Have you suddenly lost your sense of smell/taste in the last 2 months?

☐ Yes

☐ No

1. How would you rate your sense of smell at its worst?

| 0 | 1 | 2 | 3 | 4 | 5 | 6 | 7 | 8 | 9 | 10 |
| --- | --- | --- | --- | --- | --- | --- | --- | --- | --- | --- |

I have nothing at all Completely normal

1. How would you rate your sense of taste (salt/sweet/sour/bitter/savory) at its worst?

| 0 | 1 | 2 | 3 | 4 | 5 | 6 | 7 | 8 | 9 | 10 |
| --- | --- | --- | --- | --- | --- | --- | --- | --- | --- | --- |

I have nothing at all Completely normal

1. If you have suffered symptoms, were they…

☐ Mild and I stayed at home

☐ Moderate with coughing/fever/etc.

☐ Severe – led to hospitalisation

☐ Other:

1. Did your sense of smell/taste disappear…

☐ Before any other symptoms

☐ After other symptoms

☐ It is the only symptom

☐ Not applicable

1. What other symptoms have you experienced?

☐ Nasal blockage

☐ Runny nose

☐ Nasal irritation/burning

☐ Distortion of smell (bad smell when it should be good)

☐ Bad smell (when there isn’t one present)

☐ Fever

☐ Cough

☐ Diarrhoea

☐ Shortness of breath

☐ Fatigue

☐ Other:

1. If you suffered loss of smell/taste, did it…

☐ Return completely

☐ Return partially

☐ It hasn’t yet returned

1. If you suffered loss of smell/taste, how long did it last for (in days)?
2. Any other information you would like to add?
3. If you have had anosmia, we would like to follow you up. Please indicate that you are happy for one of the research team to contact you.

☐ Yes

☐ No

☐ Not applicable

1. Enter your email address below for contact (in case you are happy for us to contact you) – not required.
2. You may also like to participate in this global survey: https://sites.google.com/view/gcchemosensr/
3. Which hospital are you working at?
4. Does your work involve direct patient contact?

☐ Yes

☐ No (eg. Admin work only)

*Follow Up Questionnaire*

1. Date of survey completion
2. Please confirm that you have completed this survey previously.

☐ Yes

☐ No

1. Please provide us with your email address so that we can link your answers to the original survey.
2. What is your age?
3. What is your gender?
4. Have you been tested for Covid-19 Coronavirus?

☐ Yes and positive

☐ Yes and negative

☐ No but suspected positive

☐ No and no current suspicion

1. Has your sense of smell and/or taste now recovered?

☐ Yes, completely

☐ Yes, partially

☐ No

☐ Not applicable

1. Have you noticed any of the following?

☐ Distortion of smell (things smell differently to what you expect)

☐ Feeling like you are smelling something when there is no smell present

☐ Changes in the sensations of burning, cooling or tingling in your nose or mouth

☐ None of the above

1. How would you rate your sense of smell now?

| 0 | 1 | 2 | 3 | 4 | 5 | 6 | 7 | 8 | 9 | 10 |
| --- | --- | --- | --- | --- | --- | --- | --- | --- | --- | --- |

I have nothing at all Completely normal

1. How would you rate our sense of taste (salt/sweet/sour/bitter/savoury) now?

| 0 | 1 | 2 | 3 | 4 | 5 | 6 | 7 | 8 | 9 | 10 |
| --- | --- | --- | --- | --- | --- | --- | --- | --- | --- | --- |

I have nothing at all Completely normal

1. Since you completed the first survey, have you…

☐ Self-isolated at home

☐ Needed treatment in hospital yourself

☐ Continued to work as normal

☐ Other:

1. If you told us that loss of smell/taste was your only symptom, did other symptoms arise after you completed the last survey?

☐ Yes

☐ No

☐ Not applicable

1. Would you be interested in participating in a study investigating loss of sense of smell in relation to COVID-19 infection?

☐ Yes

☐ No

**Supplemental Material 2.** Combined Questionnaire

1. Date of survey completion
2. What is your age group?

☐ Under 30

☐ 31 – 40

☐ 41 – 50

☐ 51 – 60

☐ Over 60

1. What is your gender?

☐ Male

☐ Female

☐ Non-binary

☐ Prefer not to say

1. What is your ethnic background?

☐ Prefer not to answer

☐ White (English/ Welsh/ Scottish/ Northern Irish/ British/ Irish/ Gypsy/ Irish Traveller / any other White background)

☐ Mixed/ Multiple ethnic groups (White & Black Caribbean, White & Black African, White & Asian, and any other Mixed/ Multiple ethnic background

☐ Asian/ Asian British (Indian)

☐ Asian/ Asian British (Pakistani)

☐ Asian/ Asian British (Bangladeshi)

☐ Asian/ Asian British (Chinese)

☐ Asian/ Asian British (any other Asian background)

☐ Black/ African/ Caribbean/ Black British (African, Caribbean, any other black/ African/ Caribbean background)

☐ Other ethnic group (Arab, any other ethnic group)

1. Have you been tested for Covid-19 Coronavirus?

☐ Yes and positive

☐ Yes and negative

☐ No but suspected positive

☐ No and no current suspicion

1. Have you

☐ Self-isolated at home

☐ Needed treatment in hospital yourself

☐ Continued to work as normal

☐ Other:

1. Have you suddenly lost your sense of smell/taste in the last 3 months?

☐ Yes

☐ No

1. How would you rate your sense of smell at its worst?

| 0 | 1 | 2 | 3 | 4 | 5 | 6 | 7 | 8 | 9 | 10 |
| --- | --- | --- | --- | --- | --- | --- | --- | --- | --- | --- |

I have nothing at all Completely normal

1. How would you rate your sense of taste (salt/sweet/sour/bitter/savory) at its worst?

| 0 | 1 | 2 | 3 | 4 | 5 | 6 | 7 | 8 | 9 | 10 |
| --- | --- | --- | --- | --- | --- | --- | --- | --- | --- | --- |

I have nothing at all Completely normal

1. Did your sense of smell/taste disappear…

☐ Before any other symptoms

☐ After other symptoms

☐ It is the only symptom

☐ Not applicable

1. If you suffered loss of smell/taste, how long did it last for (in days)?
2. What other symptoms have you experienced?

☐ Nasal blockage

☐ Runny nose

☐ Nasal irritation/burning

☐ Distortion of smell (bad smell when it should be good)

☐ Bad smell (when there isn’t one present)

☐ Fever

☐ Cough

☐ Diarrhoea

☐ Shortness of breath

☐ Fatigue

☐ Other

1. If you suffered symptoms, were they…

☐ Mild and I stayed at home

☐ Moderate with coughing/fever/etc.

☐ Severe – led to hospitalisation

☐ Other:

1. Any other information you would like to add?
2. Which hospital are you working at?
3. Does your work involve direct patient contact?

☐ Yes

☐ No

1. If you have had a loss of sense of smell (anosmia), we would like to follow you up. Please indicate that you are happy for one of the research team to contact you.

☐ Yes

☐ No

☐ Not applicable

1. Enter your email address below for contact – in case you are happy for us to contact you.
2. Has it been 4 weeks or more since you lost your sense of smell/taste?

☐ Yes

☐ No

1. Has your sense of smell and/or taste now recovered?

☐ Yes, completely

☐ Yes, partially

☐ No

1. Have you noticed any of the following?

☐ Distortion of smell (things smell differently to what you expect)

☐ Feeling like you are smelling something when there is no smell present

☐ Changes in the sensations of burning, cooling or tingling in your nose or mouth

☐ None of the above
